# Transcription-dependent silencing of inducible convergent transgenes in transgenic mice

**DOI:** 10.1186/1756-8935-3-3

**Published:** 2010-01-19

**Authors:** Fernando J Calero-Nieto, Andrew G Bert, Peter N Cockerill

**Affiliations:** 1 Experimental Haematology, Leeds Institute of Molecular Medicine, University of Leeds, St James's University Hospital, Leeds LS9 7TF, UK; 2 Department of Haematology, Cambridge Institute for Medical Research, Wellcome Trust/MRC Building, Hills Road, Cambridge CB2 0XY, UK; 3 Human Immunology, Centre for Cancer Biology, SA Pathology, Adelaide 5000, Australia

## Abstract

**Background:**

Silencing of transgenes in mice is a common phenomenon typically associated with short multi-copy transgenes. We have investigated the regulation of the highly inducible human granulocyte-macrophage colony-stimulating-factor gene *(Csf2) *in transgenic mice.

**Results:**

In the absence of any previous history of transcriptional activation, this transgene was expressed in T lineage cells at the correct inducible level in all lines of mice tested. In contrast, the transgene was silenced in a specific subset of lines in T cells that had encountered a previous episode of activation. Transgene silencing appeared to be both transcription-dependent and mediated by epigenetic mechanisms. Silencing was accompanied by loss of DNase I hypersensitive sites and inability to recruit RNA polymerase II upon stimulation. This pattern of silencing was reflected by increased methylation and decreased acetylation of histone H3 K9 in the transgene. We found that silenced lines were specifically associated with a single pair of tail-to-tail inverted repeated copies of the transgene embedded within a multi-copy array.

**Conclusions:**

Our study suggests that epigenetic transgene silencing can result from convergent transcription of inverted repeats which can lead to silencing of an entire multi-copy transgene array. This mechanism may account for a significant proportion of the reported cases of transgene inactivation in mice.

## Background

The introduction of transgenes into the germline of animals and plants has become commonplace. Nevertheless, it remains difficult to routinely direct efficient and persistent expression of small transgenes in transgenic mice. One of the major problems facing transgenic mouse model systems is that transgenes frequently undergo silencing [1-3]. Transgene silencing is most prevalent when genes integrate as multi-copy transgene arrays [2,4,5] and it is normally accompanied by DNA methylation and formation of a repressive chromatin environment [1-3]. However, transgene silencing is not restricted to small multicopy transgenes, because even large single copy bacterial artificial chromosome (BAC) transgenes are susceptible to silencing associated with position effect variegation [6].

In mammalian transgenic models, it has been established that transgene silencing is mediated by epigenetic mechanisms but it is unclear why tandem arrays of transgenes are so prone to silencing. There is evidence that convergent transgenes are silenced more strongly than tandem repeats, but it has also been observed that any repeated arrangement of transgenes can undergo some degree of silencing [7]. Although the mechanism of transgene silencing in mammalian cells is not fully understood, in plants there is also evidence that silencing occurs predominantly at sites where transgene arrays include inverted repeated DNA sequences [4,8-12]. In such instances it has been suggested that transgene silencing occurs via convergent transcription and the synthesis of palindromic RNAs and RNAi. Until recently, such a mechanism was not believed to exist in mammalian cells. However, there is now substantial evidence that targeting of siRNAs to active genes can indeed direct epigenetic silencing in mammalian cells via histone deacetylation and methylation [13-18].

**Figure 1:**
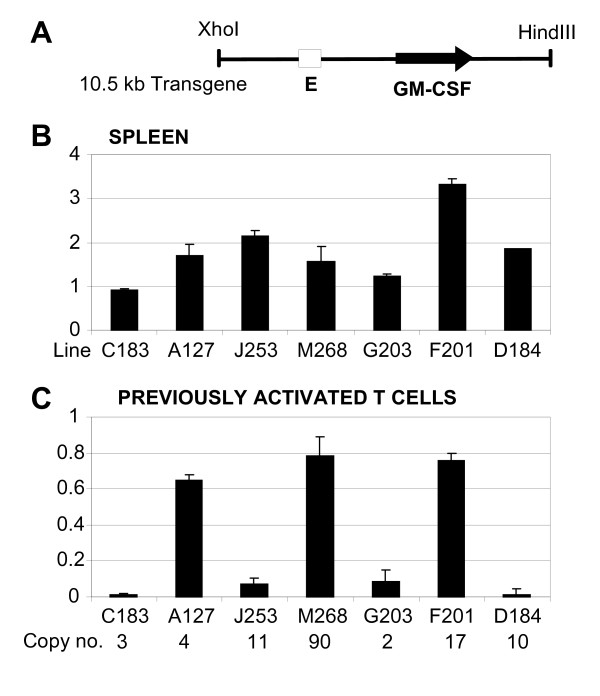
**Human granulocyte-macrophage colony-stimulating factor (GM-CSF) transgene expression in T lineage cells**. (A) Map of the 10.5 kb Xho I-Hind III fragment of the human GM-CSF locus used to make transgenic mice. (B and C) ELISAs of human GM-CSF expression in cultures of transgenic splenocytes (B) and previously activated T blast cells (C). The relative level of human GM-CSF expression per gene copy is expressed as (human GM-CSF/mouse GM-CSF)/(number of copies of human GM-CSF transgene/two copies of mouse GM-CSF transgene). In each case cells were stimulated for 15 h with 20 ng/ml PMA and 1 μM A23187. Error bars indicate standard error. The number of copies of the transgene is displayed below each line in panel C.

We have created a transgenic mouse model that allows us to study the induction of transgene silencing within multi-copy transgene arrays in mammals. We have generated lines of human granulocyte- macrophage colony-stimulating-factor (GM-CSF) transgenic mice in which the GM-CSF gene (also called *Csf2*) is tightly regulated and highly inducible [19,20]. We have demonstrated that a 10.5 kb segment of the human GM-CSF locus (Figure 1A) is sufficient to reproducibly support a level of induction of GM-CSF expression per gene copy that is indistinguishable from the endogenous mouse GM-CSF locus [19,21]. In splenic T cells that have not been previously activated, there is no evidence of silencing of transgenes in any of the many GM-CSF transgenic lines we created, even though all of the lines contain multiple copies (up to 90) of the locus [19,21]. However, in this study we show that after one episode of transcription activation, the transgene is transcriptionally silenced in a subset of lines associated with tail-to-tail inverted repeats. Furthermore, we show that this is an epigenetic phenomenon because the silenced transgenes contain the typical chromatin marks associated with long-term silencing and lose the ability to recruit RNA polymerase II. This suggests that convergent transcription may represent a common fundamental epigenetic mechanism of gene silencing in transgenic mice.

## Results

### Correct regulation of GM-CSF transgenes in splenic T lineage cells

Our group previously created transgenic mice from a 10.5 kb Xho I - Hind III segment of DNA carrying the human GM-CSF gene and all of the elements required for its correct regulation in vivo (Figure 1A) [19]. Transgene expression was assayed in activated spleen cells where the predominant GM-CSF-expressing cells are T cells. We demonstrated that this transgene is expressed in activated spleen cells in an inducible copy number-dependent fashion at levels equivalent to the endogenous mouse GM-CSF gene in 10 out of 11 independent lines of transgenic mice.

Here we reanalysed GM-CSF transgene activity in a variety of T lineage cell populations in seven of the GM-CSF transgenic mouse lines (C183, A127, J253, M268, G203, F201 and D184) carrying from two to 90 copies of the transgene (Figure 1). Freshly isolated spleen cells were stimulated for 8 h with a combination of the phorbol ester phorbol myristate acetate and the calcium ionophore A23187 (PMA/I) to directly activate T cell receptor signalling pathways. Human and mouse GM-CSF expression levels were then measured by ELISA. After correction for gene copy number, transgene expression in all lines was efficiently induced to a level approximately one to three times that of the endogenous mouse GM-CSF gene (Figure 1B). These data confirm that the 10.5 kb transgene contains sufficient information to correctly regulate GM-CSF gene expression in a position-independent and copy-number-dependent manner.

### Transcription-dependent silencing of GM-CSF transgenes in previously activated T cells

In order to investigate transgene regulation in a defined cell type we cultured actively dividing T cells derived from the spleen. In order to induce proliferation, spleen cells were activated in culture for 2 days in the presence of the lectin Concanavalin A (ConA), which activates receptor signalling pathways and induces cytokine gene transcription. We confirmed that the GM-CSF gene was, indeed, induced under these conditions (data not shown). These rapidly dividing cells were further cultured for several cell cycles in the presence of IL-2 and in the absence of any cytokine gene-inducing agent for an additional 2 days. This procedure reliably generates cultures of ~98% pure T cells that have undergone blast cell transformation from inactive non-dividing resting T cells to rapidly proliferating T cells (T lymphoblasts).

After the 2 days of culture in IL-2, the T cells had undergone at least five cell divisions since the cessation of the initial episode of activation. Cells were then restimulated with phorbol ester and calcium ionophore (PMA/I). In lines A127, M268 and F201 the transgenes were efficiently induced to levels roughly equivalent to the endogenous mouse GM-CSF gene (Figure 1C). In contrast, transgene activity was greatly reduced for lines J253 and G203 and almost non-existent for lines C183 and D184. This indicated that a profound degree of gene silencing had taken place subsequent to the initial activation of the spleen cells. Note that splenocytes are comprised primarily of quiescent cells that have had no recent history of activation by agents that induce cytokine gene transcription, whereas the cultured T cells were recently activated and had transiently expressed the transgenes. These observations suggest that a single episode of transcriptional activation is, therefore, sufficient to induce heritable transgene silencing in a specific subset of lines that persists for at least several cell cycles. The results are in marked contrast to our parallel studies carried out on several lines that contain 130 kb GM-CSF transgenes which do not undergo silencing in T blast cells in any instances (Mirabella et al, in press). This change in expression pattern is unlikely to be influenced by any change in the proportions of CD4 and CD8 positive T cells during culture because these two populations show essentially identical levels of GM-CSF expression (Mirabella et al, in press).

In summary, we have defined three transgenic lines where regulation appears to be correct under all conditions and four lines that are correctly regulated until exposed to a cycle of transcriptional activation. In order to further explore the basis of this post-activation-specific silencing we selected two correctly regulated lines (A127 and M268) and two lines that are susceptible to induction of silencing (J253 and D184) for further study. In addition to having similar activities in spleen cells, these four lines are also known to be expressed at equivalent inducible levels in peritoneal myeloid cells [21].

### Transgene silencing occurs at the transcriptional level

**Figure 2:**
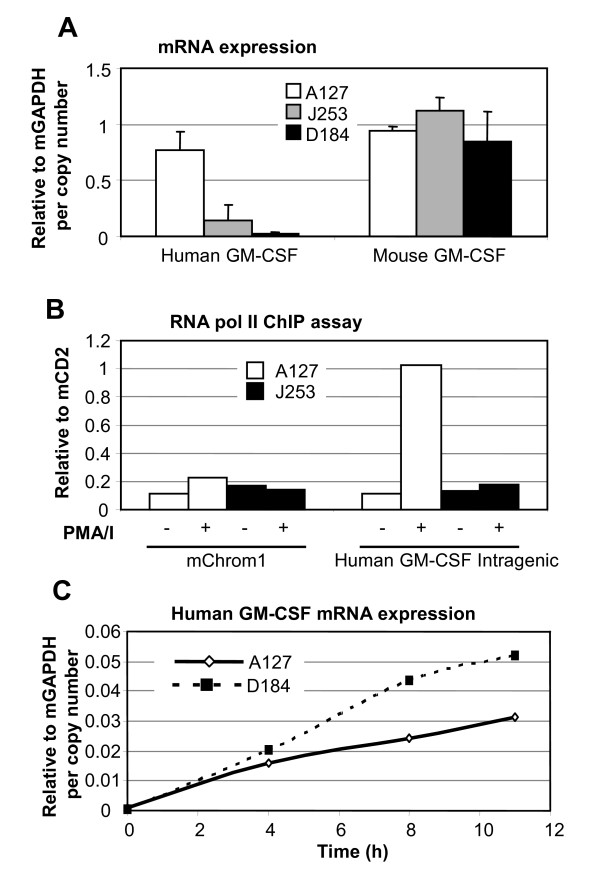
**Transcriptional gene silencing in a subset of lines**. (A) Real time polymerase chain reaction (PCR) analyses of human and mouse granulocyte-macrophage colony-stimulating factor (GM-CSF) mRNA expression in transgenic T cells. GM-CSF expression in spleen-derived T cells activated for 2 days in the presence of ConA, and then cultured for 2 days in the absence of ConA and re-stimulated for 4 h with 20 ng/ml phorbol myristate acetate (PMA) and 2 μM A23187. Values are expressed relative to mouse glyceraldehyde phosphate dehydrogenase (GAPDH) and divided by transgene copy number. Error bars indicate standard deviation. (B) chromatin immunopreciptation (ChIP) assay of the elongating Ser 2 phosphate form of RNA polymerase II performed on T lymphoblasts prepared from mouse lines A127 (open boxes) and J253 (closed boxes) before and after stimulation for 4 h with 20 ng/ml PMA and 1 μM A23187. This panel depicts a representative experiment but we have obtained similar results in an independent analysis of lines M268 and D184. (C) Time course of human GM-CSF induction in spleen cells activated with 20 ng/ml PMA and 2 μM A23187.

The above findings were verified by real time polymerase chain reaction (PCR) analysis of human and mouse GM-CSF mRNA levels in T cells stimulated for 4 h with PMA/I. As above, these cells had been first activated for 2 days in the presence of ConA and then cultured for an additional 2 days after the removal of ConA. In lines J253 and D184, human GM-CSF mRNA induction was dramatically reduced compared to line A127, whereas mouse GM-CSF mRNA was expressed at similar levels in all lines (Figure 2A). This analysis also indicated that the homologous mouse GM-CSF gene does not undergo silencing in parallel with the human GM-CSF gene in lines J253 and D184.

In order to confirm that these results reflect ongoing transcription in the nucleus, and not just steady state levels of cytoplasmic mRNA, we performed chromatin immunoprecipitation (ChIP) assays to measure levels of the Serine-2-phosphorylated elongating form of RNA polymerase II within the coding region of the human GM-CSF transgenes. Specific ChIP DNA levels were measured before and after stimulation with PMA/I by real time PCR using primer sets located within intron 2 of the GM-CSF gene. No recruitment of the elongating form of RNA polymerase II could be detected in lines D184 and J253, whereas a high level of inducible recruitment was observed in lines A127 (Figure 2B) and M268 (data not shown). This result confirms that silencing takes place at the transcriptional level.

Next, we tried to determine whether transgene silencing was a rapid process, becoming established coincident with the initial transcription initiation, or whether it was a longer process that might even require DNA replication. In order to study short term events we performed a time course of stimulation of splenocytes with PMA/I for up to 11 h (Figure 2C). Over this time period, human GM-CSF mRNA was induced with similar kinetics in both lines A127 and D184 with no major decrease in transgene activity in D184 at the later time points. As GM-CSF mRNA is highly unstable, this suggests that silencing takes more than 11 h to become firmly established. Interestingly, this time course is consistent with the reported kinetics of siRNA-mediated epigenetic gene silencing [18].

### Transgene silencing occurs at the level of chromatin structure

**Figure 3:**
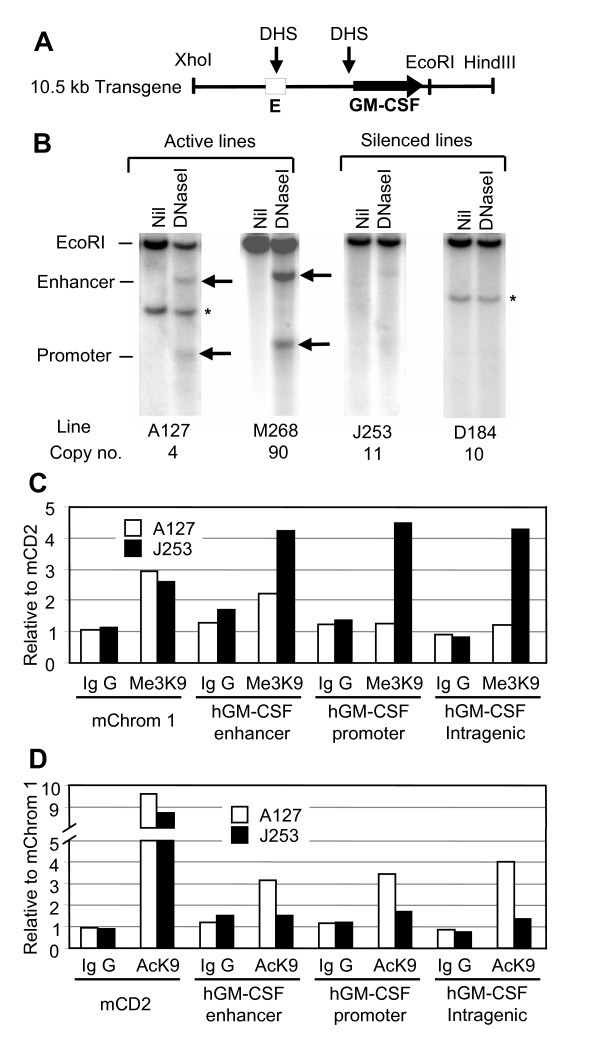
**Chromatin structure of granulocyte-macrophage colony-stimulating factor (GM-CSF) transgenes**. (A) Map of the human GM-CSF transgene, showing the positions of the GM-CSF gene and enhancer (E) and the locations of DNase hypersensitive sites (DHSs). (B) Analysis of DHSs in T lymphoblasts stimulated for 8 h with 20 ng/ml phorbol myristate acetate (PMA) and 2 μM A23187. Nil represents intact genomic DNA cleaved with Eco RI and the right hand lane in each panel is an Eco RI digest of DNA derived from DNase I-digested nuclei. The asterisks represent incomplete copies of the transgene. Mapping was performed using a Sal I-Eco RI fragment located within the GM-CSF gene. (C and D) Chromatin immunoprecipitation assays (ChIP) of tri-methylation of histone H3 K9 (C) and acetylation of histone H3 K9 (D) with non-specific immunoglobin G (IgG) as a ChIP antibody control. Assays were performed on cultured T cells previously activated with ConA, prepared from mouse lines A127 (open boxes) and J253 (closed boxes) after stimulation for 4 h with 20 ng/ml PMA and 2 μM A23187. Shown here are single representative experiments for lines A127 and J253 that have been replicated using lines M268 and D184, respectively.

The GM-CSF locus contains inducible DNase I hypersensitive sites (DHSs) located at the promoter and enhancer (Figure 3A) [19]. In order to explore epigenetic mechanisms of transgene silencing, DHSs were mapped in cultured T cells within the four chosen lines of mice. In activated T cells from lines A127 and M268 the predicted DHSs formed within the transgenes (Figure 3B). However, the formation of these DHSs was almost abolished in line J253 and completely abolished in line D184. These changes in chromatin structure paralleled the expression data where the transgenes were almost completely silenced in line D184 but incompletely silenced in line J253 (Figures 1C and 2A).

In order to further explore the basis for this chromatin-mediated gene silencing, we performed ChIP assays to measure levels of acetylation and tri-methylation of histone H3 K9 within the enhancer, promoter and coding region of the transgenes. These chromatin modifications have been widely associated with either active (acetylation) or long-term silenced (tri-methylation) regions. The ChIP assays indicated that the enhancer, promoter and coding regions of the silenced J253 transgenes were each heavily modified by trimethylation of histone H3 K9, whereas the active A127 transgenes were not significantly modified (Figure 3C). For an inactive control we also assayed a non-transcribed gene desert region of mouse chromosome 1 (mChrom1) and found that this was equally methylated in both A127 and J253, although not as strongly as the silenced transgenes. ChIP assays also showed that silencing in J253 was accompanied by a decrease in acetylation of K9 in all three regions of the transgenes in comparison with A127 (Figure 3D). Additional ChIP assays were performed on cultured T cells prepared from lines M268 and D184. These assays produced results with values very similar to those shown in Figures 3C and 3D, with D184 transgene silencing being accompanied by decreased acetylation and increased tri-methylation of H3 K9 relative to M268 (data not shown).

### Transgene silencing is not mediated by DNA methylation

**Figure 4:**
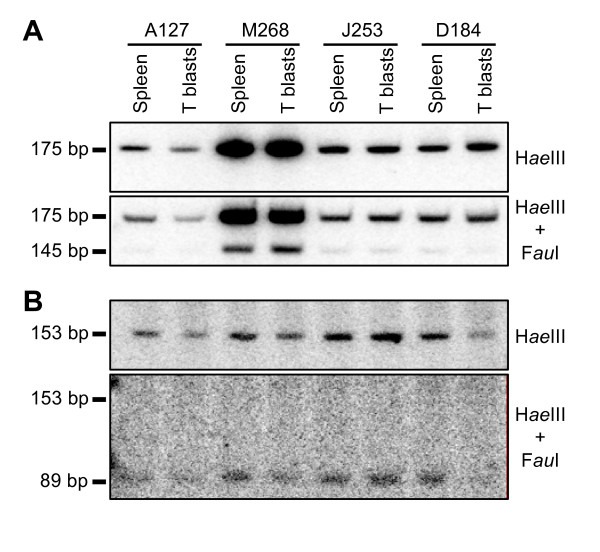
**DNA methylation analysis**. (A) Filter hybridization analysis of Fau I cleavage of Hae III-digested genomic DNA prepared from transgenic splenocytes and T lymphoblasts. Fau I recognises the Sp1 site in the human granulocyte-macrophage colony-stimulating factor (GM-CSF) promoter and is located within a 175 bp Hae III fragment which was used as the probe. DNA was purified from either whole spleen or from cultured T cells previously activated with ConA (T blasts). 5 μg of each DNA sample was digested with either just Hae III, or Hae III plus Fau I. After electrophoresis, DNA was electrophoretically transferred from a polyacrylamide gel to a nylon membrane and hybridized with ^32^P-labelled probes. (B) Reprobing of the same membrane used in (A) with a DNA fragment encompassing a CG island within the mouse adenine phosphoribosyltransferase gene.

Changes in gene expression and histone modification patterns are often, but not always, associated with changes in DNA methylation. However, the GM-CSF promoter region contains very few CG sequences that might influence gene expression. Only one CG exists within the -114 to +28 region that constitutes the defined GM-CSF promoter and this is located within the Sp1 site at -70 [22]. In order to investigate whether DNA methylation is involved in the silencing of GM-CSF transgenes we employed methylation-sensitive restriction enzymes and direct DNA hybridisation analysis of genomic DNA to determine the methylation status of the Sp1 site in GM-CSF promoter. In order to measure any changes in the degree of methylation upon silencing, DNA was purified for all four lines from both spleen, which should not be silenced, and ConA-treated cultured T cells (T blasts). Genomic DNA was digested with Hae III in the presence and absence of the methylation-sensitive enzyme Fau I which cleaves the CCCGC sequence at the Sp1 site only if it is not methylated. Hae III alone generates a 175 bp genomic DNA fragment spanning the Sp1 site, whereas Fau I creates a 145 bp sub-fragment. Products were analysed by polyacrylamide gel electrophoresis and filter hybridisation (Figure 4A). Unexpectedly, all samples were equally highly resistant to Fau I digestion of the Sp1 site. This suggested that the Sp1 site was almost fully methylated in both the spleen and the T blast cells, in both the active and the repressed lines, and that there was no change in status upon silencing. We confirmed that the low level of Fau I cleavage was not due to under-digestion because a parallel control analysis of a Fau I site located within a non-methylated CG-island revealed complete cleavage (Figure 4A). However, the significance of methylation of the single CG that exists in the promoter is unclear because DNA methylation does not necessarily interfere with Sp1 binding or function [23]. DNA methylation may not, in fact, play much of a role in the regulation of GM-CSF expression because relatively few CG sequences exist anywhere in the GM-CSF locus. Parallel analyses of 4 Hpa II sites located from 153 to 2091 bp downstream of the transcription start site suggested that similar high levels of DNA methylation existed throughout the GM-CSF gene in T cell DNA prepared from all four transgenic lines and also in primary human T cells (data not shown). Others have similarly shown that the GM-CSF gene is comprised of methylated DNA in T cells [24]. It is also now evident that it is common to find that the bodies of active genes with low CG densities are in fact methylated [25].

### Silenced transgenes are associated with inverted convergent repeats

**Figure 5:**
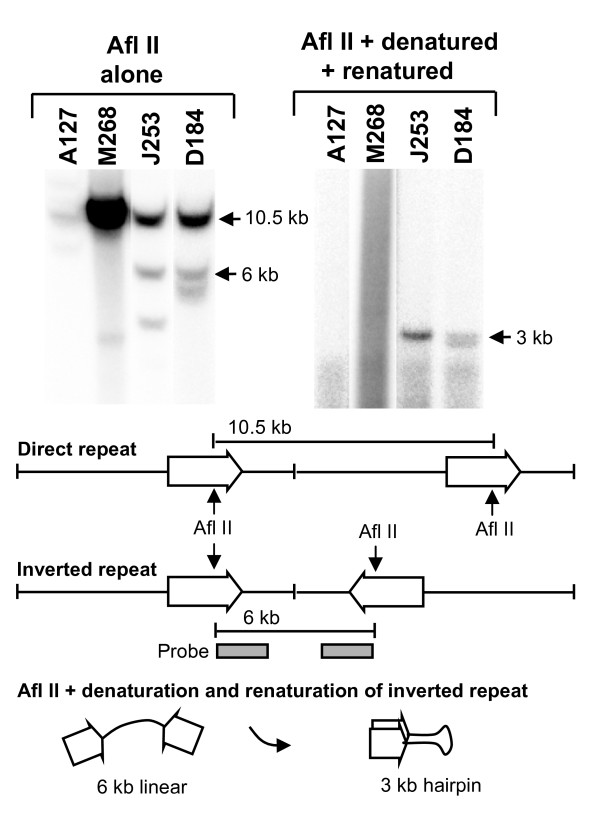
**Analysis of inverted repeats in granulocyte-macrophage colony-stimulating factor (GM-CSF) transgenic mice**. Southern blot hybridisation analysis of Afl II-digested DNA purified from 4 lines of transgenic mice, as indicated. The left hand panel is an analysis of double stranded DNA digested with Afl II and probed with a 0.83 kb Afl II-Eco RI fragment of the GM-CSF gene. The right hand panel is an analysis of the same samples after denaturation at 99°C and rapid renaturation at 66°C before electrophoresis. The schematic below illustrates potential products and the probes employed to detect them. Sample preparation is described in the methods section.

Silencing of GM-CSF transgenes appeared to be transcription-dependent and one potential mechanism of transgene silencing is convergent transcription. In multi-copy transgenes this could lead to the formation of palindromic RNA and siRNAs, which have the potential to direct localised epigenetic silencing [13-16]. Although transgenes typically integrate as head-to-tail copies within multi-copy arrays, silencing could result from convergent transcription of any less commonly encountered tail-to-tail copies of transgenes. In order to determine whether silenced lines do contain convergent gene repeats, we performed a Southern blot hybridization analysis of Afl II-digested DNA from each line, using a probe at the 3' end of the transgene to identify restriction enzyme fragments diagnostic of convergent inverted repeats. Afl II cuts once within the transgene and will generate a 10.5 kb fragment from head-to-tail repeats, a 6 kb band from tail-to-tail repeats and a band of unknown size spanning the site where the 3' end of the transgene array has integrated into the mouse genome (Figure 5). Significantly, the diagnostic 6 kb inverted repeat was present in both of the silenced lines J253 and D184, and absent in A127 and M268 (Figure 5, left hand panel). Each lane also has at least one additional band that most probably represents the Afl II fragment spanning the site of integration. In order to exclude the possibility that the 6 kb Afl II bands represent either fragmented copies of the transgene or site of integration products, we repeated this analysis with three additional restriction enzymes and obtained similar findings, which suggests that the 6 kb Afl II bands are, indeed, inverted repeats (data not shown). Densitometric quantitation of band intensities indicated that each silenced line had just one pair of convergently transcribed transgenes (data not shown).

In order to further confirm that the 6 kb Afl II products are true palindromes, we employed the tactic of denaturing the digested DNA and rapidly renaturing the single stranded products before loading the DNA on a gel for Southern blot hybridisation analysis (Figure 5, right-hand panel). Under these conditions, the hybridization kinetics do not favour the slow reannealing of separate strands of homologous DNA but do favour the rapid formation of hairpin structures from palindromes. This analysis revealed the existence of the expected 3 kb renatured hairpin product of the palindromic 6 kb Afl II fragment only in the silenced lines J253 and D184.

### Transgene junctions are transcribed

In order to determine whether there was the potential for convergent transcription to generate palindromic hairpin RNA species, we performed several analyses to determine whether transcription occurred in the vicinity of 3' or 5' junctions between individual copies of transgenes. Transgenic line C42 [21], which does not undergo silencing, is derived from a 130 kb Age I DNA fragment spanning the entire IL-3/GM-CSF locus and including about 35 kb of DNA downstream of the GM-CSF gene. In this line, it is unlikely that the GM-CSF transgene could be subjected to anti-sense transcription arising from adjacent copies. We therefore chose this line as a more appropriate model to search for evidence of sense strand transcription proceeding from the GM-CSF gene and up to and beyond the 3' Hind III site.

**Figure 6:**
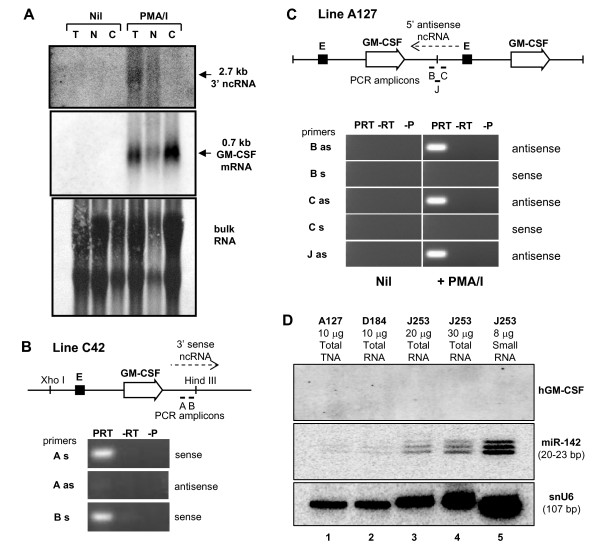
**Analysis of non-coding RNA in granulocyte-macrophage colony-stimulating factor (GM-CSF) transgenic mice**. (A) Northern blot analysis of GM-CSF mRNA expression probed with 1.3 Sal I-Eco RI gene fragment, and 3' non-coding RNA expression probed with a 1.0 kb Bgl II-Hind III fragment. The lower panel shows methylene blue staining of RNA. (B and C) Strand-specific reverse transcription and PCR amplification of regions A, B, C, and J using the primers listed in Table 2, and T cell RNA prepared before and after stimulation. Line C42 contains 6 copies of a 130 kb segment of the GM-CSF locus whereas line A127 contains 4 copies of a 10.5 kb Xho I-Hind III fragment of the GM-CSF locus. PCR reactions employed reverse transcriptase and PCR primers (PRT), primers but no reverse transcriptase (-RT), or reverse transcriptase without PCR primers. Bars depict PCR amplicons. (D) Analysis of siRNAs in transgenic T cells. Either total RNA (lanes 1 to 4), or fractionated small RNA (lane 5) was subjected to polyacrylamide gel electrophoresis, transferred to nylon membranes and hybridised with the probes described in the methods section. RNA was prepared from cells either 1 (lanes 2 and 3), 3 (lane 3) or 7 (lanes 4 and 5) days after initial stimulation of spleen cells with ConA, which was removed after the first 2 days. The upper panel encompasses the region of the filter where siRNAs should migrate (~20-25 bp).

For the first RNA assay we performed Northern blot hybridization analysis of total cellular RNA (T), nuclear RNA (N) and cytoplasmic RNA (C) prepared from cultured T cells from line C42 (Figure 6A). We detected the predicted inducible 0.7 kb GM-CSF mRNA transcript in all three fractions of RNA (middle panel, Figure 6A). We also detected an inducible 3' 2.7 kb nuclear RNA species with a 1.0 kb probe encompassing a Bgl II - Hind III fragment comprising the 3' end of the transgene (top panel, Figure 6A). Such a transcript could potentially span the 3' boundary of the transgene and might be expected to exist as a palindrome at sites of inverted repeats. This transcript could potentially represent a read-through transcript from the GM-CSF gene.

We next performed strand-specific reverse transcription of activated C42 T cell RNA, followed by PCR in order to detect transcripts in two adjacent regions just inside the 3' Hind III site (amplicons A and B, Figure 6B). This revealed the presence of sense strand, but not anti-sense strand transcripts, within 40 bp of the Hind III site which probably proceed even further past this point. If the same predicted pattern of transcription occurs in lines J253 and D184, then this would, indeed, generate palindromic hairpin RNA species that could direct gene silencing.

We then examined lines A127, J253 and D184 for the presence of this 3' species of RNA. Curiously, in line A127, in contrast to line C42, we were not able to detect sense but did detect anti-sense strand transcripts downstream of the GM-CSF gene with primer set B (Figure 6C). These transcripts 3' of the gene could potentially arise from anti-sense transcripts originating from the GM-CSF enhancer located within the next downstream copy of the transgene. In support of this view, our group has detected RNA polymerase II in association with the GM-CSF enhancer in activated T cells from transgenic line C42 in ChIP assays (Mirabella et al, in press). We then examined line A127 in order to determine whether there are anti-sense transcripts potentially originating from the GM-CSF enhancer that can traverse the 5' boundary of the transgenes. This could also generate palindromic RNA at inverted repeats. We again used strand-specific reverse transcription and PCR using primers either upstream (B) or downstream (C) of the Xho I site that defines the 5' boundary of each transgene copy (Figure 6C). Note that probe B is, in fact, downstream of the GM-CSF gene and is designed to detect transcription within adjacent copies. We also employed primers that span the junction between transgenes (J). As before, in this analysis we detected anti-sense, but not sense transcripts with both the 5' and the junction primer sets, and also with additional primers closer to the enhancer (data not shown). This suggests that the enhancer does, indeed, direct transcription into neighbouring copies of the transgene. We interpret these observations as an indication that 5' anti-sense transcription from the enhancer can suppress the competing 3' sense strand transcription from the gene in head-to-tail repeats of the transgene. However, in the case of tail-to-tail repeats, there would be no such suppression because there is no adjacent downstream copy of the GM-CSF enhancer. These transcripts were absent prior to the induction of the expression of the gene with PMA and calcium ionophore, indicating that they are inducible (data not shown). We have, however, been unable to detect any of these transcripts in the silenced lines J253 and D184, very likely due to the fact that GM-CSF transcription in these lines is shut down after the act of epigenetic silencing (data not shown).

Taken together, these data suggest that a single inverted copy of a transgene, embedded within a tandem array, does indeed have the potential to generate palindromic RNAs at both boundaries of the transgene, which could be the trigger for epigenetic silencing.

### Small interfering (si) RNAs are not detected in silenced transgenes

It has been well documented that double stranded RNAs can lead to production of small RNAs after their processing. In order to search directly for the presence of GM-CSF siRNAs in lines J253 and D184 we have performed an extensive series of Northern blot analyses. One such example is shown in Figure 6D. In this analysis we have assayed either total cellular RNA, or a sample enriched for low molecular weight RNA, from previously activated T cells prepared from lines A127, D184 and J253. Although we can easily detect other specific small RNA species, we were unable to detect any GM-CSF siRNAs (Figure 6D). We have now completed many exhaustive attempts at detecting a GM-CSF siRNA in the silenced lines using various T cell preparations at different stages of blast cell transformation, using a range of probes and have failed to find such an RNA anywhere in the GM-CSF locus (data not shown).

In mammalian cells, siRNAs are thought to be able to direct epigenetic gene silencing by recruitment of Argonaute 1 or 2 [14,15]. However, we have also been unable to detect any recruitment of either Argonaute 1 or 2 in ChIP assays (data not shown). Hence, although it is clear that GM-CSF transgene silencing is occurring at the level of transcription, and is an epigenetic phenomenon, the precise mechanism of silencing remains unknown.

## Discussion

### Multi-copy transgenic loci are not necessarily silenced

There is strong evidence that multi-copy transgenes are prone to silencing, and at least one example in mammalian cells where the removal of all but one copy of a transgene is sufficient to relieve such silencing [2]. However, it remains unclear why transgene silencing has only been observed in a proportion of the cases where multi-copy transgene arrays have been studied. In apparent contrast to some previous studies, we did not observe silencing of tandemly repeated head-to-tail transgenes and we found that a 90 copy transgene array was expressed just as efficiently per gene copy as a four copy transgene array (Figure 1) [19]. It is generally assumed that transgenes integrate in transgenic mice as direct repeats and, for the most part, they do. However, we have shown that transgenes can also integrate into arrays at low frequency as inverted repeats. Significantly, we have shown that transcriptional silencing takes place in the subset of lines that contains inverted repeats of convergent transgenes, which suggests that these facts may be directly related. There is at least one other known example of a transgenic mouse line where transgenes have integrated as inverted repeats and are expressed at an unexpectedly low efficiency [26]. We also suggest that the mere act of convergent transcription is unlikely to be sufficient since we have clearly demonstrated that the GM-CSF locus generates both 3' sense and 5' anti-sense transcripts and so there must logically be a point in our head-to-tail multi-copy transgenic lines where these opposing transcripts converge without this process leading to silencing of these loci.

While we have presented one possible explanation for multi-copy transgene silencing, this is not the only mechanism by which multi-copy transgenes can be silenced. For example, in *Drosophila *there is evidence of pairing of homologous sequences such that even head-to-tail repetitions of transgenes can lead to heterochromatin formation and transgene silencing [27]. However, this is in contrast to what we have observed in mouse T cells.

### Transgene silencing is an epigenetic phenomenon

We have also shown that maintenance of GM-CSF transgene silencing in T cells is accompanied by the formation of a repressive chromatin environment that resembles heterochromatin. As suggested by our ChIP assays, the formation of this repressive chromatin environment is most probably driven by modifications that include deacetylation of histones followed by trimethylation of histone H3 K9. Heritable silencing of the transgenes in lines J253 and D184 was accompanied by the absence of DHSs. This suggests that not just the inverted copies of the locus, but the entire transgene array of 10 or 11 copies, was converted to condensed inactive chromatin. This effect is specific for the transgenes since the expression of the endogenous copy of the mouse GM-CSF gene was not affected. Others have reported that transgene silencing associated with position effect variegation is also accompanied by a loss of DHSs within regulatory elements [6]. As we have not examined expression at the single-cell level, we are unable to state whether GM-CSF transgene silencing involves reduced expression in all cells or position effect variegation.

DNA methylation does not appear to be important in the silencing process because the GM-CSF promoter and transcription unit were highly methylated even in the fully active transgenes and the level of methylation did not change after silencing. Similarly, it has been reported by others that gene silencing is not always associated with changes in DNA methylation [28]. Furthermore, there is evidence from a chicken cell model system that, during the process of transgene silencing, transgene transcriptional inactivation and histone hypoacetylation precede the onset of DNA methylation [29]. Furthermore, even active genes can be targeted for DNA methylation [25].

### Convergent transcription is followed by transcriptional silencing

This study has established that inducible GM-CSF transgene silencing occurs at the transcriptional level and strongly suggests that it is a transcription-dependent process. This may be a more common phenomenon than is currently appreciated because it is not very common for investigators to examine the orientation of the transgenes in studies such as this. Furthermore, many of the transgenes that have been investigated are constitutively active and preclude any analysis of inducible transcription-dependent silencing. In one study of the constitutively active α-globin enhancer transgene in erythroid cells it was found that a greater than 100 copy array was silenced but a single copy transgene in the same locus was not [2]. However, in this instance it is not known if silencing was accompanied by the presence of inverted repeats, and the timing of silencing could not be examined. In another study, this issue was examined more directly by inserting pairs of transgenes in various orientations and it was found that the convergent arrangement led to the most pronounced silencing of the transgenes [7]. However, in this study, any arrangement of two transgenes was more susceptible to silencing than a single transgene and silencing occurred despite the presence of the SV40 polyadenylation/transcription termination elements downstream of the transgenes. Hence, silencing of convergent transcription units does not necessarily always involve an overlap of the actual transcripts.

The generation of long regulatory RNA transcripts by enhancers and locus control regions (LCRs) has been extensively reported [30-32]. If transgene silencing is transcription-dependent, then the nature of the promoter and enhancer elements in transgenes may also have a large bearing on whether or not a transgene becomes silenced. Hence, the positioning of enhancers or LCRs upstream or downstream of transgenes, close to transgene boundaries, may also increase the likelihood of silencing at inverted repeats [30-32]. Furthermore, there is direct evidence that inclusion of the β-globin LCR in transgenes can, in fact, lead to transgene silencing via a mechanism that it is likely to involve transcriptional interference [33]. Similar to our findings, establishment of silencing in this model system was a slow process, and, once silenced, the transgenes retained a stable epigenetic imprint that maintained silencing [34]. In this instance, gene silencing was dependent upon the orientation of the transgene, but not the LCR, and it was apparent that silencing was triggered by activation of transcription within non-coding flanking sequences [33,34]. Hence, this could also involve an siRNA-dependent mechanism, whereby the LCR induces transcripts within flanking sequences in the opposite orientation to convergent transcripts directed by the transgene promoter [33]. Such a mechanism could, in principle, be an alternative explanation for our findings. Furthermore, via this mechanism, even single copy transgenes are prone to silencing, depending upon the site and orientation of integration.

As mentioned above, it has already been established that transgene silencing in plants can occur via convergent transcription and the synthesis of palindromic RNAs and RNAi. Furthermore, it is possible to relieve silencing at palindromic sequences in plants by deleting one copy of the inverted repeated sequence [8]. On the other hand, non-coding RNA-mediated silencing is reported to be able to occur independently of the RNAi pathway [35,36]. The fact that we have failed to detect any small RNAs derived from the silenced transgenes precludes us from making any statements one way or the other as to whether GM-CSF transgene silencing involves siRNA. However, we suggest that the formation of palindromic RNAs may be central to the silencing process. Furthermore, several studies have led to the model whereby siRNA may pair with nascent transcripts at gene loci in order to direct epigenetic silencing via Argonaute family protein-dependent processes thought to direct recruitment on the histone methyl transferase Suv39H1 and HDAC-1 [13-15,17,18]. This would be consistent with our evidence of reduced acetylation and increased methylation of histone H3 K9 within silenced transgenes. Our findings are also consistent with observations that siRNA-mediated epigenetic silencing requires up to 3 days to induce and is stable for over a month [18].

The role of non-coding transcripts in the GM-CSF locus is not clear, but misdirected transcripts may well have unwanted consequences. The human genome is widely transcribed, but only a small proportion of the transcribed RNAs codes for a protein product [37]. Although the existence of non-coding RNAs (ncRNAs) is very well known [38], in general their functions are not well understood yet. Over the last few years, the function of the ncRNAs has been subjected to exhaustive studies and its important regulatory role has become evident [39]. They have been linked to many processes such as the silencing of imprinted genes [40-42] and gene regulation [43]. In transgenes, there is plenty of potential for these ncRNAs to have suppressive effects, and so the precise organisation of transgenic loci is all important.

## Conclusions

Our study provides new insights regarding the stability of gene expression in transgenic assays. We have clearly shown that transgene silencing does not correlate with copy number, but, more importantly, it seems to correlate with the configuration of the transgene. Our results show that silencing is heritable through several cell cycles and occurs at the level of chromatin. We provide data demonstrating that either read-through or other non-coding sense-strand transcripts can be detected downstream of the GM-CSF gene. Potentially, these transcripts could form palindromes in tail-to-tail configurations and may, thus, cause silencing of the associated transgenic gene locus. This mechanism, involving either read-through transcription or enhancer-derived transcription, could be responsible for a significant proportion of the many other cases where silencing of transgenes has been reported. This phenomenon may also be restricted to short transgenes lacking significant 3' flanking sequences able to buffer the coding regions, as we see no such silencing with 130 kb BAC transgenes and there is some acceptance that large BACs are, in general, less prone to silencing in transgenic mice. These findings point to the possibility of including efficient transcription terminators downstream of the transcribed regions of transgenes as a means of lessening the incidence of transgene silencing. Although there is some evidence that this approach will not always work [7], others have found that inclusion of an efficient polyadenylation and transcription pause site can diminish gene silencing directed by transcriptional interference [44].

## Methods

### Transgenic mice

The transgenic mouse lines A127, M268, J253, D184, C183, G203 and F201 are all previously described lines created from a 10.5 kb Xho I-Hind III segment of the human GM CSF gene [19]. Note that line J253 was previously referred to as J10 in the original publication [19,21] and that, since the original characterization of these mice, the transgene copy number in each line has been recalculated by Southern blot hybridization analysis in a more recent publication [19,21]. The revised copy numbers are displayed in Figure 1C.

All of the studies involving animals followed internationally recognized guidelines and were peer-reviewed and approved by the appropriate local institution and national ethical review bodies. All studies performed at the University of Leeds were approved by the University of Leeds Ethical Review Committee and were also reviewed and granted a Project Licence by the UK Home Office (approvals PPL 40/2471 and PPL 40/3086). Previous published studies that generated mice used here were approved by the Institute of Medical and Veterinary Science Animal Ethics Committee in Adelaide, Australia (approval 5/97).

### Cells

Single cell suspensions of splenocytes were prepared by gently crushing spleens and extracting the free cells with culture medium, as previously described [19]. Previously activated proliferating T cells were prepared from spleens by stimulation for 2 days with 2 μg/ml concanavalin A (ConA) in order to induce proliferation, followed by 2 days of culture in the absence of ConA and in the presence of 10 U/ml mouse IL-2, as previously described [19]. When re-stimulated, these cultured T cells were incubated for 4 h, in the presence of 20 ng/ml phorbol 12-myristate 13-acetate (PMA) and 2 μM calcium ionophore A23187 (I), in order to activate T cell receptor signalling pathways that normally induce GM-CSF expression.

### GM-CSF ELISAs

Human and mouse GM-CSF protein levels in cell culture media were measured by ELISA (R & D Systems, Oxfordshire, UK) after 15 h of stimulation with 20 ng/ml PMA and 1 μM A23187. The relative activity per gene copy was calculated as the ratio of (human GM-CSF/mouse GM-CSF)/(transgene copy number/2).

### Real time PCR analyses of gene expression

Total cellular RNA was isolated using Trizol (Invitrogen, Renfrew, UK) according to the manufacturer's instructions. Human and mouse GM-CSF and mouse glyceraldehydes phosphate dehydrogenise (GAPDH) mRNA levels were measured by real time PCR analysis of cDNA primed using olio (dot), as previously described [20], using the primer sets listed in Table 1.

**Table 1:** Real time polymerase chain reaction (PCR) primers.

Real time PCR primers	
h GM-CSF mRNA	CACTGCTGCTGAGATGAATGAAA
	
	GTCTGTAGGCAGGTCGGCTC

m GM-CSF mRNA	ATGCCTGTCACGTTGAATGAAG
	
	GCGGGTCTGCACACATGTTA

m GAPDH mRNA	TGGTGAAGCAGGCATCTGAG
	
	TGTTGAAGTCGCAGGAGACAAC

ChIP primers	

h GM-CSF enhancer	GGAGCCCCTGAGTCAGCAT
	
	CATGACACAGGCAGGCATTC

h GM-CSF promoter	TGTCGGTTCTTGGAAAGGTTCA
	
	TGTGGAATCTCCTGGCCCTTA

h GM-CSF intron 2 (+ 469 to 533 bp)	ATGGCAGTCACATGAGCTCCTT
	
	TGAAGTGACCCCCACTTTACCA

m CD2 promoter	CTCTCTCCTTCCCCATCTCTACCT
	
	CAACCTGAACCACGTGTCTTTC

m chromosome 1 (mChrom1)	CATAGATGAAGCTGCCACATAGGT
	
	GTGGGCAAGGACAAAGCATTA

GM-CSF, granulocyte-macrophage colony-stimulating factor; GAPDH, glyceraldehyde phosphate dehydrogenase

### Strand-specific PCR analysis of ncRNAs expression

Strand specific reverse transcription and PCR was performed using the primers listed in Table 2 for regions located approximately 0.5 kb 5' (A) or 0.1 kb 5' (B) of the Hind III site defining the 3' end of the transgene, 0.1 kb 3' of the Xho I site that defines the 5' end of the transgene (C) or were designed to span the Hind III/Xho I junction between head-to-tail copies of transgenes (J).

**Table 2:** Non-coding RNA strand-specific reverse transcription and polymerase chain reaction primers.

Linker	CGACTGGAGCACGAGGACAC
A as RT	CGACTGGAGCACGAGGACACCAGAGCCCTGAACCTGTTTC

A as R	ATGTAAACCTTCGTTATGTGATG

A s RT	CGACTGGAGCACGAGGACACATGTAAACCTTCGTTATGTGATG

A s Rev	CAGAGCCCTGAACCTGTTTC

B as RT	CGACTGGAGCACGAGGACACTGACACAGGTGGCTATCCTCTGGAA

B as Rev	CCTGAGAATCTCTGAATCCCCA

B s RT	CGACTGGAGCACGAGGACACCCTGAGAATCTCTGAATCCCCA

B s Rev	CACAGGTGGCTATCCTCTGGAA

J as RT	CGACTGGAGCACGAGGACACAGGCTGAGGTCATGGACTT

J as Rev	GCCCTAAAGCCTCCCCACC

C as RT	CGACTGGAGCACGAGGACACTGATGAGAAGGCTGGGAGGCTG

C as Rev	GGTTTTCTGTTTTGGCTTGCT

C s RT	CGACTGGAGCACGAGGACACTGACAGCCTCCCAGCCTTCTCA

C s Rev	GGGGTGGGGAGGCTTTAG

CDNA was synthesised with Thermo-X Reverse Transcriptase from Invitrogen (Renfrew, UK). All RNA samples were pre-treated with DNase I to remove contaminating traces of genomic DNA. Each cDNA reaction contained 500 ng RNA and 2 μM specific oligonucleotide primers in 20 μl. In order to increase the strand specificity, we attached a linker sequence (Table 2) to the 5' end of each specific primer. Three reactions per cDNA synthesis were set up: one with reverse transcriptase and with specific primer; one with reverse transcriptase but without primer (to control for potential endogenous priming); and one without reverse transcriptase but with primer (to control for potential DNA contamination) (designated as PRT, -P and -RT, respectively, in Figure 6B). After the RNA denaturation step at 65°C, tubes were kept at 60°C throughout the procedure in order to prevent endogenous random priming. For cDNA synthesis, reactions were incubated at 60°C for 30 min with 1 μl of Thermo-X Reverse transcriptase. The reverse transcriptase was then inactivated by incubating at 90°C for 5 min. The primers used with reverse transcriptase are listed in Table 2 and are designated as 's RT' or 'as RT' depending on whether they were designed to detect sense or anti-sense transcripts, respectively.

Subsequent to the reverse transcription step, to amplify cDNA PCRs were performed with Invitrogen native Taq DNA polymerase (Invitrogen, Renfrew, UK). Five per cent of the specific cDNA synthesis product obtained above was used as a template in each reaction. PCR was performed using one cycle at 95°C for 3 min, 31 cycles of 95°C 30 s, 60°C 30 s, 72°C 1 min 30 s and one cycle of 72°C for 5 min. For each PCR reaction, one primer containing the linker sequence was used in place of the RT primers used above, together with specific reverse direction primers (denoted as Rev) as listed in Table 2.

### ChIP assays

ChIP assays were performed essentially as published in reference [21]. Upstate antibodies: 5 μg anti-acetyl histone H3 K9 (07-352), 2 μg anti-tri-methyl histone H3 K9, 5 μg of rabbit polyclonal IgG (12-370). Abcam antibody: 2 μg of anti-RNA polymerase II Ser-2 phosphate (ab5095-100). Real time PCR was used to determine the amount of each gene-specific amplicon present. All values were determined from standard curves using input DNA purified from sonicated chromatin. ChIP data was normalised by expressing the amount of each specific DNA precipitated as a ratio with the values obtained with either an inactive region of mouse chromosome 1 (mChrom1) or the promoter region of the expressed CD2 gene. Primer sequences are shown in Table 1.

### Analysis of DNase I hypersensitive sites

DNase I hypersensitive sites (DHSs) were mapped within T cell nuclei as previously described [20,45] from a Eco RI site at the 3' end of human GM-CSF gene, using a 1.2 kb Sal I-Eco RI fragment of the gene as a probe to detect DHSs in the GM-CSF gene promoter and enhancer.

### DNA methylation analysis

Genomic DNA was digested with Hae III in the presence and absence of Fau I, separated by polyacrylamide gel electrophoresis, electrophoretically transferred to a Hybond N nylon membrane and hybridized with ^32^P-labelled DNA probes. Probe DNA templates were prepared by PCR and were designed to recognise either the 175 bp Hae III fragment within the human GM-CSF promoter or the 153 bp Hae III fragment of a CG island located within the mouse adenine phosphoribosyltransferase gene.

### Detection of palindromes

In order to detect inverted repeat sequences, 5 μg of DNA purified from each transgenic line was digested with Afl II and analysed by Southern blot hybridisation analysis using a 0.83 kb Afl II-Eco RI fragment of the GM-CSF gene as a probe.

In order to create and detect hairpin structures from DNA fragments containing inverted repeats, DNA digested with Afl II was heated at 99°C for 10 min, gradually cooled over ~15 min to 66°C, incubated at 66°C for 15 min, cooled to room temperature and then analysed by Southern blot hybridisation as above.

### Detection of small RNAs

Total RNA was purified from cells as above for the gene expression analyses. Small RNAs were prepared from total RNA by precipitation of large RNAs with 5% polyethylene glycol 8000 and 0.5 M NaCl, followed by addition of 0.1 volume of 3 M Na Acetate, pH 5.5 and precipitation of small RNAs with 3 volumes of ethanol. RNA was analysed by electrophoresis on 15% polyacrylamide gels containing 7 M urea, followed by electrophoretic transfer to Hybond N+ membranes and fixation using 0.12 J/cm^2 ^ultraviolet light. Membranes were sequentially hybridised with either a 3.2 kb Hind III-Eco RI fragment encompassing the entire GM-CSF gene and promoter, or previously published DNA oligo-nucleotide probes complementary to either mouse U6 small nuclear RNA (snU6) (TGTGCTGCCGAAGCGAGCAC) or mouse micro RNA 142 (miR-142) (CTAGTGCTTTCTACTTTATG) [46]. Hybridizations were performed for 2 hours in RapidHyb buffer (Amersham) at 50°C, and membrane washes were performed in 300 mM NaCl, 30 mM Na citrate, 1 mM Na pyrophosphate, 0.2% Na dodecyl sulphate, pH 7 for 30 min at 50°C.

## Abbreviations

BAC: bacterial artificial chromosome; ChIP: chromatin immunoprecipitation; DHS: DNase I hypersensitive sites; GAPDH: glyceraldehyde phosphate dehydrogenase; GM-CSF: granulocyte-macrophage colony-stimulating factor; LCR: locus control region; ncRNA: non-coding RNA; PCR: polymerase chain reaction; PMA/I: phorbol 12-myristate 13-acetate plus calcium ionophore A23187; siRNA: small interfering RNA.

## Competing interests

The authors declare that they have no competing interests.

## Authors' contributions

FJC-N and PNC designed the study and prepared the manuscript. FJC-N carried out most of the experimental work. AGB performed ELISAs on stimulated spleen cells. PNC performed DNase I analyses and assays of palindromes. All authors read and approved the manuscript.
